# Blood pressure outcomes at 18 months in primary care patients prescribed remote physiological monitoring for hypertension: a prospective cohort study

**DOI:** 10.1038/s41371-024-00904-7

**Published:** 2024-03-06

**Authors:** Stephen D. Persell, Lauren Anthony, Yaw A. Peprah, Ji Young Lee, Jim Li, Hironori Sato, Lucia C. Petito

**Affiliations:** 1 Division of General Internal Medicine, Department of Medicine, Feinberg School of Medicine, Northwestern University, Chicago, IL USA; 2 Center for Primary Care Innovation, Institute for Public Health and Medicine, Feinberg School of Medicine, Northwestern University, Chicago, IL USA; 3 Northwestern Medical Group Quality and Patient Safety, Northwestern Memorial Healthcare, Chicago, IL USA; 4 Strategic Clinical RD Department, Technology Development HQ, Omron Healthcare, Co., Ltd., Kyoto, Japan; 5 Data Analytics Department, Technology Development HQ, Omron Healthcare, Co., Ltd., Kyoto, Japan; 6 Division of Biostatistics, Department of Preventive Medicine, Feinberg School of Medicine, Northwestern University, Chicago, IL USA

**Keywords:** Hypertension, Disease prevention

## Abstract

This pragmatic matched cohort study using EHR data extended the follow up to 18 months for BP outcomes comparing individuals prescribed remote patient monitoring (*n* = 288) and temporally-matched controls (*n* = 1152) from six primary care practices. After 18 months, the RPM-prescribed cohort had greater BP control < 140/90 mm Hg (RPM cohort: 71.5%, control cohort: 51.9%, *p* < 0.001) and lower systolic BP (131.6 versus 136.0 mm Hg, *p* = 0.004) using office and home measurements. BP control at 18 months assessed by office measurements only was also higher in the RPM group (62.2% versus 51.9%, *p* = 0.004).

**TO THE EDITOR:**

Self-measurement of out-of-office blood pressure (BP) combined with tele-monitoring interventions has been shown to improve BP control in clinical trials [1–5]. Previously, we conducted two pilot studies at six primary care practices where remote patient monitoring (RPM) was made available to clinicians [6, 7]. We then compared patients who were prescribed RPM to propensity-score-matched cohorts of controls from those same clinics [8]. Here we report the extension of that pragmatic matched cohort study to include 18 months of follow up.

The intervention cohort consisted of all patients prescribed RPM for blood pressure and the control cohort, matched 4 to 1 was created from temporally-matched patients who did not receive a RPM prescription. We compared BP-related outcomes, including hypertension control, SBP achieved, and antihypertensive medication intensification. This study was conducted at primary care practices (three in Chicago, three suburban) within the Northwestern Medicine health system. Patients were prescribed RPM between November 18, 2020 and August 14, 2021. RPM patients and their matched controls were followed for 18 months after the RPM prescription. All study data, including data used for identifying the comparison cohorts and outcome data, were abstracted the system’s electronic health record (EHR) data. The study was approved by the Northwestern University Institutional Review Board. Methods used for matching and details of the outcomes assessment have been preveiously described [8]. At the start date for each RPM-prescribed patient or matched controls, all patients were required to be aged 65 to 85 years, have Medicare or Medicare Advantage insurance, and have had at least one office or telehealth visit in the preceding year. Outcome ascertainment occurred at 18 months after the index date for all patients. The primary effectiveness outcome was the Controlling High Blood Pressure performance measure—National Quality Forum Measure 0018 (NQF0018)—most recent BP < 140/90 mm Hg within 12 months of the measurement date [9, 10]. Patients without a BP measurement in the preceeding 12 months did not meet the measure. Secondary outcomes included most recent primary care in-office systolic BP and antihypertensive medication intensification. The absolute number of antihypertensive medication intensifications were determined from the EHR medication list. Net increases in antihypertensive medications were considered present if the number of antihypertensive drug classes added or with a dose increased minus the number of classes discontinued or with a dose decreased was greater than zero. Senistivity analyses evaluated Controlling High Blood Pressure and systolic BP using only primary care office BPs. Among patients prescribed RPM, we measured intensity of use (mean readings per 30 days over the months used during the 18 month observation period), and duration of use (time from first reading to a reading with no subsequent reading for 30 days). Generalized linear models were used to estimate differences between RPM-prescribed patients and matched controls in mean systolic BP (identity link) and differences in log-odds for categorical variables (logit link); a random effect for patient was included to account for correlation between observations on the same control who was matched to multiple RPM-prescribed patients.

The resulting cohorts were 288 RPM patients and 1152 matched controls (Table 1). After 18 month of follow up, the median duration of use of RPM was 14 months (interquartile range 7 to 17 months). During months when RPM was used, the median number of blood pressure readings per month was 25 (IQR 13 to 39). Baseline rates of Controlling High Blood Pressure were low in both groups (35.4% RPM cohort, 39.2% controls, *p* = 0.24). The differences in blood pressure outcomes observed at 18 months between the RPM and control cohorts were similar to those seen at earlier intervals [8]. Compared with controls, a greater proportion of RPM-prescribed patients had Controlled High Blood Pressure at 18 months, 71.5% vs. 51.9%, Odds Ratio 2.3 (95% confidence interval 1.7 to 3.1; *p* < 0.001). RPM-prescribed patients had lower systolic BP compared to controls at 18 months: 131.6 mm Hg versus 136.0 mm Hg, *p* = 0.004, Table 1). In a sensitivity analysis that included only in-office primary care BP measurements, the RPM cohort had a higher prevalence of Controlling High Blood Pressure at 18 months—62.2% vs. 51.9%, OR 1.5 (1.1 to 2.0; *p* = 0.004). Most recent office systolic BP measurements at 18 months were not significantly different, 133.6 (18.5) mm Hg for RPM cohort, 136.1 (19.1) for controls, difference −1.9 mm Hg (−4.5 to 0.7). The distributions of antihypertensive medication changes and the proportion with a net antihypertensive medication increase was similar in the two cohorts. After 18 months, 44.1% of the RPM and 44.4% of the control cohort had a net increase in antihypertensive medication therapy (*p* = 0.99).

**Table 1:** Baseline characteristics and comparison of blood pressure outcomes at 18 months in patients prescribed remote patient monitoring (RPM) compared with matched controls.

	Intervention *N* = 288	Matched controls *N* = 1152		
**Baseline characteristics**^**a**^				
Age, years, mean (SD)	73.6 (7.4)	73.8 (7.9)		
Female, *n* (%)	195 (67.7)	814 (70.7)		
Race/ethnicity, *n* (%)				
Non-Hispanic White	204 (70.8)	797 (69.2)		
Other	84 (29.2)	355 (30.8)		
SBP, mean (SD)	142.7 (19.5)	141.2 (18.7)		
DBP, mean (SD)	77.3 (9.8)	75.9 (9.5)		
Controlling High Blood Pressure	35.4%	39.2%		
**Outcomes at 18 months**	**Intervention** ***N*** = **288**	**Matched controls** ***N*** = **1152**	**Effect size (95% confidence interval)**	***P***^**b**^
Controlling High Blood Pressure, 18 months^c^	71.5%	51.9%	OR 2.3 (1.7, 3.1)	<0.001
Controlling High Blood Pressure (primary care office blood pressure only), 18 months^c^	62.2%	51.9%	OR 1.5 (1.1, 2.0)	0.004
Systolic blood pressure in mm Hg, 18 months, mean (SD)^d^	131.6 (18.5)	136.0 (19.0)	−3.9 (−6.5, −1.3)	0.004
Systolic blood pressure in mm Hg (primary care office blood pressure only), 18 months, mean (SD)^d^	133.6 (18.5)	136.1 (19.1)	−1.9 (−4.5, 0.7)	0.15
Net antihypertensive medication increase, *n* (%)	44.1%	44.4%	OR 1.0 (0.8, 1.3)	0.99

*OR* odds ratio.

^a^See reference [8] for full baseline characteristics used for matching.

^b^*p* values for differences in means or proportions were calculated from generalized linear mixed models, with a random intercept for patient.

^c^The lowest recorded BP was used if there were multiple measurements on the same date.

^d^The average daily SBP was used if there were multiple measurements on the same date. This sample underwent some attrition between baseline and follow-up: 13% of RPM-prescribed patients and 18% of matched controls for systolic blood pressure measure.

These findings extend the follow up of a previously-reported RPM intervention in hypertensive patients in routine primary care using a matching cohort design. Compared with controls, RPM-prescribed patients were more likely to meet the Controlling High Blood Pressure metric and had lower mean systolic BP after 18 months. At the 18-month time point, there was a divergence in the rate of Controlling High Blood Pressure between the cohorts using only office measurements, a difference that was not present at earlier time points. It is also notable that BP differences similar to what were observed at 3 to 12 months persisted at 18 months even though the median user only used RPM for 14 months. These findings suggest that the incorporation of RPM into the care of hypertensive patients has effects on hypertension control that may persist longer than the period of RPM use. These findings complement those from the TASMINH4 randomized controlled trail which demonstrated improved blood pressure lowering with blood pressure self-monitoring with or without a telemonitoring intervention [4] by providing outcome data from remote patient monitoring obtained in real-world United States practice settings without a protocolized treatment approach. While the methods used do not directly identify the mechanisms through which better blood pressure control was achieved, possible explanations include improved adherence to medication or health behaviors, or improved measurement accuracy in the home setting compared to office setting. While there were no differences between groups in antihypertensive medication changes overall, it is possible that remote monitoring led to more appropriate medication changes—patients with elevated home blood pressures had medication intensification while those with lower home blood pressure had dose reductions. This could be evaluated in future research.

There are inherent limitations to this study design that should be mentioned. The study was not randomized. The matched design minimized the differences between patients on the multiple characteristics included in the matching algorithm but does not account for unmeasured characteristics.

In this matched cohort study conducted with EHR data from six clinics, we observed higher prevalences of Controlling High Blood Pressure and lower systolic BP after 18 months among patients prescribed RPM in routine primary care compared with controls. However, to elucidate the mechanisms through which RPM improves BP requires additional study.

## Summary

### What is known about this topic

- Experimental hypertension management strategies using home blood pressure monitoring and specific care teams have successfully improved blood pressure but less is known about the effects of introducing remote monitoring into routine primary care.
- At 18 months of follow up, a cohort of 288 primary care patients prescribed remote physiological monitoring for blood pressure was more likely to have controlled high blood pressure compared to a propensity-score matched control cohort.

### What this study adds

- In these real-world practice settings, Medicare patients prescribed RPM persisted with use for a median of 14 months.
- Compared with controls, primary care patients prescribed remote physiological monitoring for blood pressure were more likely to have controlled high blood pressure at 18 months.

## Data Availability

A deidentified dataset will be shared with other investigators on reasonable request to the corresponding author.
